# Surgical Repair of Two Kinds of Type A Aortic Dissection After Thoracic Endovascular Aortic Repair

**DOI:** 10.3389/fcvm.2022.849307

**Published:** 2022-03-30

**Authors:** Zhou Fang, Haiyang Li, Thomas M. Warburton, Junming Zhu, Yongmin Liu, Lizhong Sun, Wenjian Jiang, Hongjia Zhang

**Affiliations:** ^1^Department of Cardiac Surgery, Beijing Anzhen Hospital, Capital Medical University, Beijing, China; ^2^Beijing Institute of Heart Lung and Blood Vessel Diseases, Beijing, China; ^3^Department of General Surgery, St Vincent’s Hospital Sydney, Darlinghurst, NSW, Australia; ^4^Faculty of Medicine, University of New South Wales, Sydney, NSW, Australia

**Keywords:** total arch replacement, frozen elephant trunk, type A aortic dissection, thoracic endovascular aortic repair, retrograde dissection

## Abstract

**Background:**

Retrograde dissection is now recognized as an important complication following thoracic endovascular aortic repair (TEVAR). The purpose of this study is to describe two different situations of TAAD after TEVAR. We will introduce the surgical methods used to repair TAAD following TEVAR at our center, and evaluate its long-term prognosis.

**Methods:**

Between January 2010 and October 2019, 50 patients who had previously received TEVAR treatment for TBAD were admitted to our center for repair of a type A aortic dissection. According to the patients’ CT angiographies and intra-operative findings, we identified two distinct groups: a retrograde group (stent-induced new aortic injury, with retrograde extension involving the ascending aorta) and an antegrade group (entry tear located in the aortic root, ascending aorta or the aortic arch, away from the edges of the stent grafts). The options for treatment of the proximal aorta were Bentall procedure (12/50, 24.0%) and ascending aorta replacement (38/50, 76.0%). All patients underwent total arch replacement (TAR) and frozen elephant trunk (FET) implantation. Survival over the follow-up period was evaluated with the Kaplan–Meier survival curve and the log-rank test.

**Results:**

The median interval time from prior TEVAR to reoperation was 187 days (IQR: 30.0, 1375.0 days). 18.0% of TAAD after TEVAR did not have any obvious symptoms at the time of diagnosis, most of which were found on routine follow-up imaging. The patients in the retrograde group were younger than those in the antegrade group (44.0 ± 9.4 vs. 51.4 ± 10.5 years, *P* = 0.012). No significant differences in the incidence of post-operative complications or mortality were noted between the two groups. The mean follow-up time was 3 years. No late death or complications occurred after one year following surgery upon follow-up. The asymptomatic survival rate one year after surgery was 90.0%.

**Conclusion:**

The TAR and FET technique was feasible and effective for complicated TAAD after TEVAR. The surgical success rate and long-term prognosis of patients undergoing the timely operation are satisfactory.

## Introduction

Thoracic endovascular aortic repair (TEVAR) was initially designed to treat disease of the descending thoracic aorta more than 20 years ago (1). With the development of hybridization techniques, complex aortic arch lesions no longer present as many challenges in the treatment of type B aortic dissection (TBAD) (2). Retrograde type A aortic dissection (rTAAD) is the most serious complication, which has a low incidence but high mortality rate (3, 4). The causes of rTAAD have been studied extensively, but for recurrent TAAD after TEVAR, clinical data are scarce and there is no consensus on treatment modalities. Here, we describe the clinical characteristics of two distinct forms of TAAD following TEVAR, introduce our center’s choice of surgical methods for this situation and report the long-term prognosis of treatment.

## Patients and Methods

### Patient Population

**FIGURE 1:**
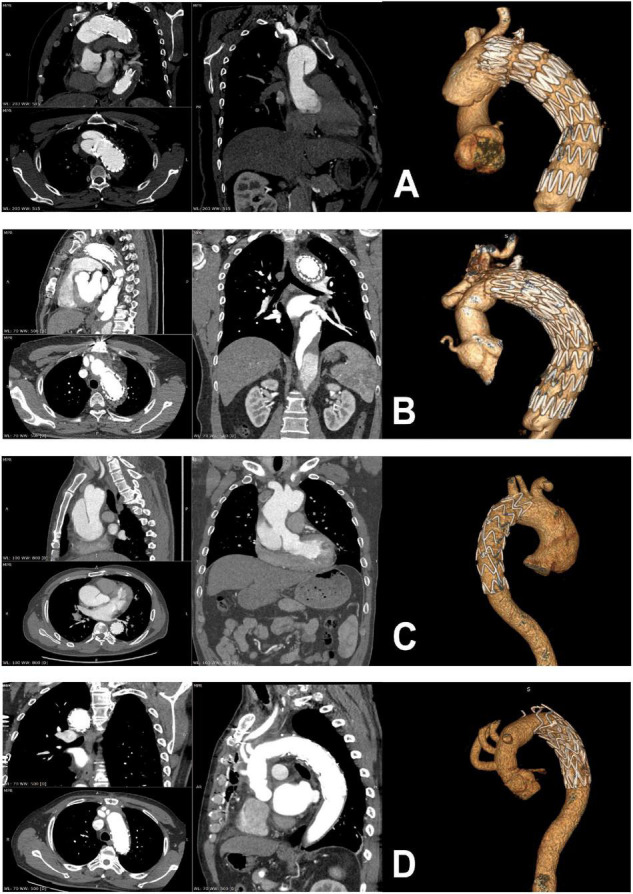
Preoperative and postoperative computed tomographic scans of patients with retrograde type A dissection **(A,B)** and antegrade type A dissection **(C,D)**.

Between January 2010 and October 2019, 50 patients that had undergone previous primary TEVAR were referred to our hospital for TAAD. We retrospectively collected the clinical data of the patients through the electronic medical record management system. By reviewing the surgical records and the imaging data and reports, we divided the 50 patients into two groups (retrograde group, *N* = 28 and antegrade group, *N* = 22) based on individual anatomical features (Figure 1). Clinical information was retrospectively collected. Basic clinical characteristics and anatomical details of pathology are summarized in Table 1.

**TABLE 1:** Baseline characteristics of participants.

Characteristic	Total (*n* = 50)	Retrograde (*n* = 28)	Antegrade (*n* = 22)	*P*-value
Age, Mean ± SD	47.2 ± 10.5	44.0 ± 9.4	51.4 ± 10.5	0.012
Male, *n* (%)	37 (74.0)	22 (78.6)	15 (68.2)	0.612
BMI, Median (IQR)	25.0 (23.7, 27.4)	25.0 (23.6, 27.9)	24.9 (23.7, 26.7)	0.799
**Pre-operative comorbidity**				
Hypertension, n (%)	39 (78.0)	23 (82.1)	16 (72.7)	0.503
Smoking, *n* (%)	25 (50.0)	16 (57.1)	9 (40.9)	0.393
Diabetes, *n* (%)	5 (10.0)	1 (3.6)	4 (18.2)	0.155
Marfan Syndrome, *n* (%)	2 (4.0)	1 (3.6)	1 (4.5)	1
Location of entry tear				<0.001
Root, *n* (%)	4 (8.0)	0 (0)	4 (18.2)	
Ascending, *n* (%)	15 (30.0)	1 (3.6)	14 (63.6)	
Arch, *n* (%)	31 (62.0)	27 (96.4)	4 (18.2)	
LVEF, Mean ± SD	61.6 ± 5.6	59.9 ± 5.6	63.9 ± 4.8	0.010
Ascending-aorta-diameter, Mean ± SD	44.7 ± 8.2	42.2 ± 6.7	47.9 ± 9.1	0.015
Aortic-sinus-diameter, Median (IQR)	40.0 (36.0, 46.0)	39.5 (35.8, 43.2)	44.0 (36.0, 47.0)	0.347
Aortic-regurgitation, *n* (%)				0.208
Mild, *n* (%)	28 (56.0)	19 (67.9)	9 (40.9)	
Moderate, *n* (%)	9 (18.0)	3 (10.7)	6 (27.3)	
Severe, *n* (%)	2 (4.0)	1 (3.6)	1 (4.5)	
**Clinical symptoms**				
None, *n* (%)	9 (18.0)	4 (14.3)	5 (22.7)	0.481
Sudden pain, *n* (%)	35 (70.0)	20 (71.4)	15 (68.2)	1

*BMI, body mass index; LVEF, left ventricular ejection fraction.*

The location of the entry tear in the retrograde group was at the proximal ending of the previous stent and closely correlated with the previous proximal landing zone. All echocardiography data were obtained pre-operatively. Interestingly, about 18.0% of patients were diagnosed as TAAD by routine follow-up imaging examination and did not experience any associated symptoms.

### Initial Thoracic Endovascular Aortic Repair Details

The vast majority of patients’ initial TEVARs were completed by other hospitals. The median interval between the primary TEVAR procedure and TAAD was 187 days (IQR: 30.0–1375 days). The details of the previous TEVAR procedure were summarized in Table 2.

**TABLE 2:** Details of previous TEVAR procedure.

Variables	Total (*n* = 50)	Retrograde (*n* = 28)	Antegrade (*n* = 22)	*P*-value
Intervals, Median (IQR)	187.0 (30.0, 1375.0)	180.0 (30.0, 832.5)	540.0 (35.2, 1810.0)	0.278
Proximal landing zone, *n* (%)				<0.01
0	1 (2.0)	1 (3.6)	0 (0)	
1	4 (8.0)	4 (14.3)	0 (0)	
2	12 (24.0)	8 (28.6)	4 (18.2)	
3	19 (38.0)	15 (53.6)	4 (18.2)	
4	14 (28.0)	0 (0)	14 (63.6)	

Since the vast majority of patients were referred from other medical centers, we could not collect specific TEVAR details, but all stent positions were able to be clarified by CT aortography and intraoperative findings on operation records.

### Operative Technique

**FIGURE 2:**
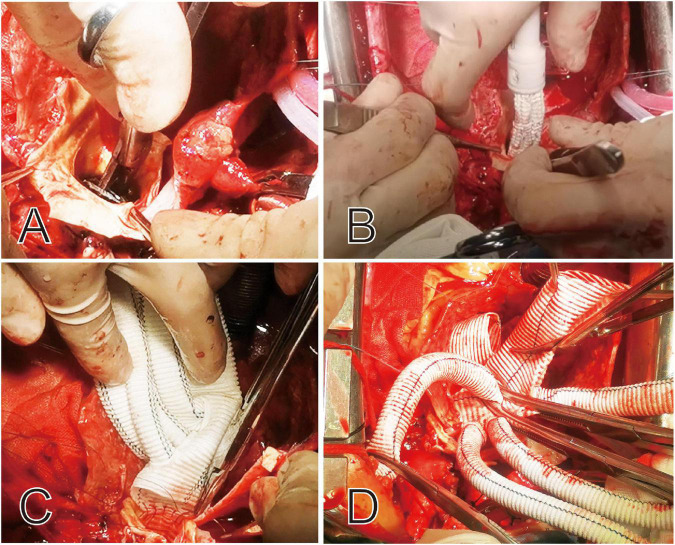
Intraoperative view of the proximal portion of the deployed stent graft and the bare springs was cut off before completion of this anastomosis **(A,B)**. The proximal ends of the stent graft and aortic wall are sewn together to tetra-furcated graft **(C,D)**.

The surgical technique, known as Sun’s procedure (total arch replacement using a tetra-furcate graft and stented elephant trunk implantation) (5), has been described in detail previously (6, 7). Specifically, right axillary artery cannulation is used for cardiopulmonary bypass (CPB) and unilateral selective antegrade cerebral perfusion under moderate hypothermic circulatory arrest at 25°C. Patients who present with an innominate artery malperfusion may not have adequate perfusion for CPB. In this case, the femoral artery is cannulated in addition to the axillary artery. Cooling was started immediately after the initiation of CPB. The proximal manipulations were carried out during the cooling period, including reinforcement of the detached commissures with ascending aorta or aortic root replacement. Once the target temperature was reached, all supra-aortic vessels were clamped and transected. The unilateral selective antegrade cerebral perfusion was initiated *via* the right axillary artery. The flow was adjusted to maintain a left radial artery pressure ≥20 mmHg. The most critical aspect of this secondary procedure was to identify any damage to the aortic wall from the proximal bare springs of the previous stents. After cutting the steel wires of the bare spring, the stented elephant trunk (Cronus, MicroPort, China) was deployed in the true lumen of the descending aorta, and TAR was performed with a tetra-furcated graft (Figure 2). Once the distal anastomosis was completed, distal reperfusion was initiated. The left common carotid artery was reconstructed first, and rewarming was then started. The ascending aorta was anastomosed to resume myocardial perfusion, followed by the left subclavian artery (LSCA), and finally the innominate artery. In some cases, we bypass the LSCA to expand the spatial range of operation and better protect cerebral perfusion, depending on anatomical location.

## Follow-Up

The primary endpoint of this study was death after the surgical repair, and the secondary endpoint was reoperation after this surgery. Follow-up was performed on all patients after discharge until the end of the study period in September 2020, or death. Survival data were obtained through clinical follow-up and phone calls. Patients who did not return to review, those whom we could not contact, and patients for whom we could not otherwise ascertain reintervention status were considered lost to follow-up. An annual computed tomography (CT) scan was recommended to detect thrombosis and obliteration of the false lumen, evaluate the sizes of the true lumen, false lumen, stented and unstented distal aortic segments, and any complications. Early mortality was defined as death at 30 days after surgery, and late mortality was defined as a death event reported at a follow-up visit other than 30 days after surgery.

## Statistical Analysis

All analyses were performed using R Statistical Software^1^ (The R Foundation) and Free Statistics analysis platform. The Kolmogorov–Smirnov test was used to assess the normal or non-normal distribution of continuous data. Normally distributed continuous data were presented as means ± SD and assessed by Student’s *t*-test. Data showing non-normal distribution were presented as median and interquartile range, and the Mann-Whitney *U*-tests were performed. Chi-squared or Fisher’s exact tests were used in categorical variables. Statistical significance is presented by *P* values. *P* value < 0.05 was considered significant. Kaplan–Meier curves were generated to assess survival data.

## Results

### Demographics and Comorbidities

In total, 78.6% of the patients who developed retrograde TAAD were men with a mean age of 44.0 ± 9.4 years. The gender composition was not significantly different from the antegrade group, but the age was significantly smaller (44.0 ± 9.4 vs. 51.4 ± 10.5 years; *P* = 0.012) than that of the antegrade group. The retrograde group had a smaller size of ascending aorta than the antegrade group (42.2 ± 6.7 vs. 47.9 ± 9.1 mm; *P* = 0.015), and the LVEF of the retrograde group was significantly lower than the antegrade group (59.9 ± 5.6 vs. 63.9 ± 4.8%; *P* = 0.010). We did not find any statistically significant differences between the two groups in terms of gender, BMI, pre-operative comorbidity, aortic regurgitation, and clinical symptoms.

### Details of the Previous Thoracic Endovascular Aortic Repair Procedure

The interval times between TEVAR and subsequent TAAD between the retrograde and antegrade groups were [180.0 (30.0, 832.5) vs. 540.0 (35.2, 1810.0) days; *P* = 0.278]. The proximal landing zone was recorded in line with the reporting standards of endovascular repair (2, 8). Table 2 outlines the clear and significant difference between the zone of proximal stent landing in the antegrade and retrograde dissection groups. 53.6% of the retrograde group had the initial proximal stent placed in landing zone 3 (<2 cm of the LSCA), and none had proximal landing in zone 4. In contrast, more than half of the antegrade group patients had proximal landing in zone 4. One patient of the retrograde group received debranching and TEVAR initially, leading to the location of the entry tear in the ascending aorta, corresponding to zone 0.

### Interoperative Data and Surgical Details

As Table 3 shows, in the choice of the surgical method, we mainly considered the anatomical location and severity of the new lesions. Chief aspects of lesion severity included the extent of the dissection tear and whether it was combined with a structural heart disorder. Sun’s procedure was chosen in all patients to deal with complicated aortic arch lesions. Because all patients had recurrent type A dissection on the basis of TEVAR treatment for type B dissection, with aortic roots involvement, resulting in aortic valve regurgitation or coronary ischemia, our center selected Bentall procedure for repair. Patients without the above conditions underwent ascending aortic replacement for the proximal aorta. Concurrent use of Bentall procedure in the retrograde and antegrade groups were 25.0 and 22.7% (*P* = 1), respectively, whilst concurrent ascending aorta replacement procedure in the retrograde and antegrade groups were 75.0 and 77.3% (*P* = 1). Across both cohorts, coronary artery bypass grafting was performed in three patients (2%) and mitral valve repair in one patient (1%). The lengths of cardiopulmonary bypass time, cross-clamped time, and circulatory arrest time were 196.1 ± 41.1, 111.2 ± 30.6, and 28.0 (22.2, 34.8) minutes, respectively. The cooling nasopharyngeal temperature was 23.9 (23.0, 24.3) °C.

**TABLE 3:** Intraoperative data.

Variables	Total (*n* = 50)	Retrograde (*n* = 28)	Antegrade (*n* = 22)	*P*-value
Operative time (h), Median (IQR)	7.5 (6.5, 8.9)	7.0 (6.0, 8.0)	8.0 (7.0, 9.9)	0.053
CPB-time (min), Mean ± SD	196.1 ± 41.1	186.9 ± 40.2	207.9 ± 40.1	0.073
ACCT (min), Mean ± SD	111.2 ± 30.6	104.0 ± 27.3	120.3 ± 32.7	0.060
DHCA-time (min), Median (IQR)	28.0 (22.2, 34.8)	28.0 (24.0, 33.0)	28.0 (21.2, 39.8)	0.696
Nasopharyngeal temperature (°C)	23.9 (23.0, 24.3)	23.9 (23.3, 24.3)	23.8 (22.9, 24.1)	0.487
**Operative technique**				
Bentall	12 (24.0)	7 (25)	5 (22.7)	1
Ascending aorta replacement	38 (76.0)	21 (75)	17 (77.3)	1
**Concomitant procedures**				
MVR	1 (2.0)	0 (0)	1 (4.5)	0.44
CABG	2 (4.0)	2 (7.1)	0 (0)	0.497

*CPB, cardiopulmonary bypass; ACCT, aortic cross-clamp time; DHCA, deep hypothermia circulatory arrest; MVR, mitral valve replacement; CABG, coronary artery bypass grafting.*

The follow-up data were available for all survivors. Follow-up was performed on all patients after discharge until the end of the study period on September 5, 2020, or death. The mean follow-up period was 3 years. As Table 4 shows, there was no statistical difference between the two groups in the major adverse events (neurological complications, respiratory failure, renal insufficiency requires dialysis, or secondary thoracotomy). All operations were successfully completed. The early mortality rate was 4.0% (2/50).

**TABLE 4:** Post-operative outcomes.

Variables	Total (*n* = 50)	Retrograde (*n* = 28)	Antegrade (*n* = 22)	*P*-value
Hospitalization-days (d), Median (IQR)	13.0 (10.0-20.0)	13.5 (9.8, 19.2)	13.0 (9.2, 24.5)	0.930
ICU-retention-times (d), Median (IQR)	2.0 (1.0-4.0)	2.0 (1.0, 3.2)	2.0 (1.0, 6.2)	0.448
Ventilator-times (h), Median (IQR)	36.0 (17.2-91.6)	31.0 (16.6, 69.4)	39.5 (18.5, 168.1)	0.358
Post-operative complications				
Neurological complications, *n* (%)	3 (6.0)	3 (10.7)	0 (0)	0.246
Dialysis, *n* (%)	3 (6.0)	1 (3.6)	2 (9.1)	0.576
Respiratory failure, *n* (%)	2 (4.0)	1 (3.6)	1 (4.5)	1
Secondary thoracotomy, *n* (%)	5 (10.0)	2 (7.1)	3 (13.6)	0.643

*ICU, intensive care unit.*

Five deaths occurred during the follow-up period, with the main causes of death after surgery listed in Table 5. Two patients with Marfan Syndrome had a thoracoabdominal aortic replacement one year after second surgery.

**TABLE 5:** Details of deaths during the follow-up period.

No	Gender	Age	PLZ	Interval times	Group	Operative procedures	Death times	Cause of death
1	Male	40	2	6 months	Retro.	Bentall + TAR + FET	37 days	Sepsis, lung infection, hepatic failure
2	Male	65	4	6 years	Ante.	Ascending aorta replacement + TAR + FET	4 days	Hemorrhagic shock, gastrointestinal bleeding
3	Female	51	2	4 years	Ante.	Ascending aorta replacement + TAR + FET	38 days	Cerebral infarction, post-operative infection
4	Male	35	1	2 months	Retro.	Ascending aorta replacement + TAR + FET	94 days	Multiple organ failure
5	Male	65	3	6 hours	Retro.	Ascending aorta replacement + TAR + FET	14 days	Liver and kidney failure

*PLZ, proximal landing zone; TAR, total arch replacement; FET, frozen elephant trunk.*

**FIGURE 3:**
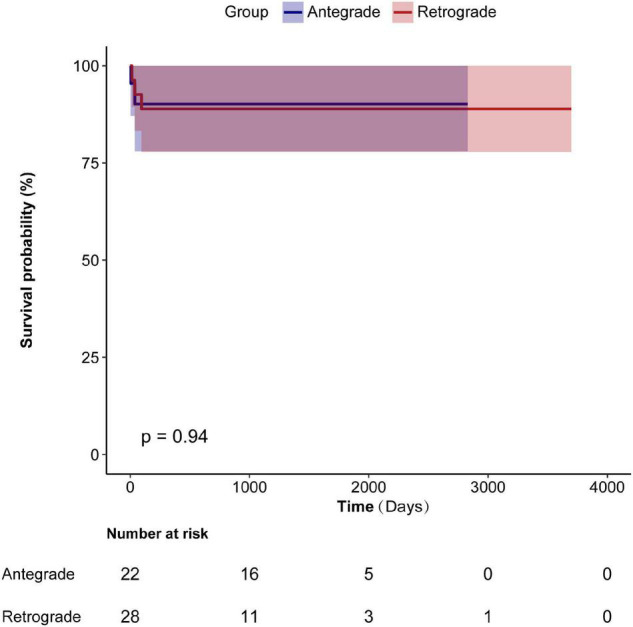
Kaplan-Meier curves show overall survival comparing retrograde group and antegrade group.

Kaplan–Meier survival curves (Figure 3) found no difference in post-operative survival between the two groups.

## Discussion

Thoracic endovascular aortic repair has been performed for more than 20 years since its first development for the treatment of aortic aneurysms (1) and dissections (9). In treating uncomplicated type B aortic dissection with appropriate anatomical conditions, current guidelines recommend an endovascular approach (10, 11). This strategy is technically effective and has the advantages of reduced trauma and quicker recovery. TEVAR promotes aortic remodeling in both acute and chronic dissections, increasing the true lumen diameter at the level of the stent graft (12). However, with the promotion of this technology and the increasing indications for hybrid surgery (2), retrograde TAAD (rTAAD) induced by previous endovascular stent-grafts is becoming increasingly recognized as the most catastrophic potential complication (3, 13). Whilst incidence of rTAAD is low, it has a high mortality (4). This can present as an early or late complication after TEVAR. It is a life-threatening complication that can be managed safely with early recognition and rapid delivery of open or hybrid repair (14). From January 2010 to October 2019, a total of 50 patients underwent surgery in our hospital for TAAD after a prior TEVAR, including antegrade dissection (*n* = 22) and retrograde dissection (*n* = 28). We selected open aortic repair in all patients, and the overall surgical outcomes were satisfactory, with repairs of both types of dissection having good long-term results upon post-operative follow-up. However, we should also note that there were two patients with rTAAD who died suddenly while waiting for surgery, and two patients that presented with rTAAD who were unconscious due to severe cerebral ischemia and their families refused surgery.

The inducement of retrograde dissection after TEVAR, and how to optimize the procedure to avoid this complication, has been widely studied.

Firstly, we believe that the TEVAR procedure is a technique that requires extensive surgical experience, which tends to be better performed in larger centers with better staffing and equipment as recommended by the guidelines (11). It’s also important that this center have a team experienced in surgical repair to address any emergency surgical needs in the case of initial procedural failure. In this study, a large number of patients were referred from primary hospitals.

Secondly, it was previously supposed that retrograde dissection cases were associated with the use of proximal bare spring stent grafts (4), however, the results of further study found that the proximal endograft configuration was not associated with any difference in the incidence of retrograde dissection (15). In our study, it was found that all patients with retrograde dissection used proximal bare spring stent-grafts, and it could be found that the steel frame punctured the aortic intima. More studies have focused on the release strategy of stents, in particular, during stent implantation across the arch, the adaptability of the stent to the aortic arch morphology must be considered. Lack of a secure landing zone, too close to the left subclavian artery, can lead to aortic mural damage due to inflexibility of the stent itself and the impact of prolonged blood flow (16). We noted that the previous proximal stent landing zones in the retrograde group were all in the unsafe zone, at-or-before zone 3, even if some were combined with surgical bypass surgery. Patients with evidence of a bird-beak configuration, after TEVAR with proximal landing zone 1 or 2, were more prone to stent-induced complications (17). A short proximal neck or steep angulation of landing zones were significantly correlated with greater incidence of TEVAR failure (18). Balloon expansion during stent release as well as the degree of oversizing of the stent itself is also an important factor responsible for retrograde dissection (15, 19). As Luehr et al. (20) pointed out, the use of stent grafts with protruding proximal bare springs and the implementation of oversizing and post-deployment ballooning should be avoided in patients undergoing hybrid arch procedures, particularly if the ascending aorta is dilated.

Thirdly, the fragility of the aortic wall and disease progression are potential contributing factors to rTAAD after TEVAR, especially in patients with Marfan syndrome, so we should avoid aortic arch stent grafting in Marfan patients (13). In our study, 2 Marfan patients developed antegrade TAAD after TEVAR treatment. After open repair, two patients underwent a later total thoracoabdominal aortic replacement after 1 year, with good outcomes at follow up. It seems that for such patients, aggressive expansion of open surgery rather than minimally invasive seems to be a better long-term option considering the likely need for further intervention.

From a hemodynamic point of view, retrograde dissection is typically more moderate in terms of blood flow velocity and false lumen perfusion than in antegrade dissection (21). Unsurprisingly, we found greater ascending aorta diameters and higher left ventricular ejection fractions in the antegrade dissection patient group. The location of primary tear in an aortic arch dissection can influence the degree of progression of the lesion. In cases involving the posterior pathway, there was generally a primary tear located in the arch or descending aorta, and cervical branch compromise was rare. However, lesions in the anterior aspect of the aortic arch were more likely to extend into the cervical branches. A false lumen pathway through the arch was strongly associated with cervical branch compromise in acute TAADs (22). Patients with primary intimal tears located in the convexity of the distal arch may be more likely to develop retrograde TAAD than patients with tears in the distal concavity (23). There was one retrograde dissection with an entry tear on the concave side, corresponding to the opening of the innominate artery, that underwent replacement of the ascending aorta alone.

Our protocol for managing the antegrade dissection groups was performed according to the standard surgical strategy for complicated TAAD, involving TAR and FET. Despite the high-risk nature of the complications, secondary open surgical or interventional procedures can be successfully performed with acceptable outcomes (24). For the treatment of retrograde dissection, many previous studies have explored this emergency situation. Zhang et al. (25) reported the use of coils and Onyx glue to create a thrombogenic environment in the retrograde false lumen, inducing thrombosis of the false lumen to enhance a proximal landing zone prior to stent graft deployment. An et al. (26) used elephant trunk implantation to treat eight retrograde TAADs. In patients who had received prior hybrid aortic repair, they successfully removed the proximal part of the stent while the distal part was left in place. Giles et al. (27) found increased propensity for secondary aortic intervention in cases with younger age, acute dissection with larger maximal aortic diameter at presentation, Marfan syndrome, when there was usage of arch vessel adjunctive procedures with the index TEVAR. Importantly, they also found that the occurrence of aorta-related reintervention does not affect survival. Dun et al. (28) described secondary open arch operations in the treatment of aortic arch disease after TEVAR, including 24 cases of retrograde type A aortic dissection, and eight cases of new antegrade aortic dissection, and found acceptable early and midterm outcomes.

However, in considering that the patient has undergone endovascular treatment, if there is then a subsequent TAAD, it may predict that the condition of the patient’s aortic wall is unsuitable for the relevant procedural elements of stent release and proximal anchoring. There will also be significant limitations in the treatment of partial arch branch vessels. We hold a positive attitude toward the selection of the total arch replacement and the effectiveness of FET in the treatment of TAAD (after TEVAR) has been confirmed in previous studies (26, 29, 30). We believe that reconstructing the stability of the whole arch system is of chief importance. We should also consider the effect of the distal end of the existing stent on vascular compliance. The key to the procedure is to establish a stable relationship between the elephant trunk stent and the previous stent. We prefer to trim the bare area at the proximal end of the previous stent and staple the frozen elephant trunk and the covered segment of the stent, as well as the vessel wall, in a “sandwich” fashion. Furthermore, in cases where the dissection does not involve the greater curvature of the aortic arch, we prefer the island anastomosis of the branching vessels to the graft, which can reduce the complexity of surgery to a certain extent and reduce the number of sutures. For cases that have undergone hybrid aortic repair in the past, our preference is to anastomose the supra-arch branches to the ascending aorta when performing ascending segment replacement.

Patients receiving open repair after prior TEVAR have good early outcomes and preservation of the stent-graft in the majority of cases (31). Canaud et al. (32) reported the results of open repair due to device failure or adverse events after TEVAR, a low mortality rate achieved despite the precarious pre-operative conditions and complex aortic pathologies of patients, including 4 retrograde type A dissections, eight stent-grafts were left *in situ*. Higashigawa et al. (33), reported some TEVAR-associated TAADs, the entry tear of 8 patients was located in the ascending aorta or the aortic arch away from the edges of stent grafts, similar to our description of the antegrade group, for the choice of their treatment modality, it is not completely consistent. However, all patients in our study were treated with TAR + FET, the 30-day mortality rate in our study was 4.0%. In comparison to the classic TAR with FET cohort in which the 30-day mortality rate was 7.8% (34), this result is satisfactory and reliable. We emphasized the purpose of our follow-up recommendation because we were concerned about the distal unstented aorta segments, especially in patients with Marfan syndrome. According to the imaging data during follow-up available to us, 32/50 patients underwent CTA in our hospital for the first time within one year after the operation, and most of them chose to undergo CTA scanning in 3 months after discharge. Through our comparison of CTA before and after discharge, we found that FET as the stable bridge between the tetra-furcate graft and the distal TEVAR stent had achieved good results. Since the proximal end of the FET is sutured on the aortic wall, when the blood flows through the FET to the inside of the distal TEVAR stent, it would not have a blow to the aorta. In 30 patients, good morphological compliances were observed. It is worth noting that during a follow-up period of about one year, we found aneurysm-like dilation of the distal aorta in two cases of Marfan patients, who received another surgical treatment in our hospital. Other patients came to our hospital outpatient consultation with imaging films, and the outpatient visit records were also an important basis for us to review the survival status.

The study is limited by its retrospective nature, small sample size, and the lack of a control group. Only eight patients in this study had their first TEVAR treatment in our hospital, so we could not know all the details about their initial TEVAR procedures. Hence, we were unable make any conclusion about the risk factors of recurrent dissection without further analysis. The mortality rate of dissection after TEVAR may be relatively low in patients who can receive timely and effective surgical treatment. Importantly, many patients with retrograde TAAD may die outside the hospital, so there is possibility of a selection bias. Most patients were located in areas that were far away from our hospital, and due to economic considering, many patients chose to review CTA in the local hospital for the first review after discharge, due to regulatory and privacy policy restrictions, we were unable to obtain their raw DICOM data. There were also some patients with poor compliance, during the follow-up process, they replied that they did not undergo the required CTA scan because they did not feel uncomfortable. Lack of some CT scan information at follow-up represent also an important limitation. Lastly, in evaluating the choice of surgical approach, we are a single central research site and would need to further expand the sample size, and expand our comparison to other novel surgical techniques.

## Conclusion

In conclusion, this study showed that the total arch replacement with frozen elephant trunk technique was feasible and effective for patients with type A aortic dissection following previous thoracic endovascular repair. The antegrade TAAD group had longer cardiopulmonary bypass time and aortic-clamp time, but in terms of long-term follow-up, there was no difference in post-operative survival between the antegrade and retrograde groups. The surgical success rate and long-term prognosis of patients undergoing timely surgery are satisfactory.

## Data Availability Statement

The raw data supporting the conclusions of this article will be made available by the authors, without undue reservation.

## Ethics Statement

The studies involving human participants were reviewed and approved by the Ethics Committee of Beijing Anzhen Hospital. The ethics committee waived the requirement of written informed consent for participation.

## Author Contributions

HZ and WJ designed the whole conception. HL, JZ, YL, and LS provided the administrative support and completed the surgical operations. ZF and HL provided the data of patients. ZF collected and assembled the data. ZF, HL, and TW contributed in writing the manuscript. All authors contributed to the article and approved the submitted version.

## Conflict of Interest

The authors declare that the research was conducted in the absence of any commercial or financial relationships that could be construed as a potential conflict of interest.

## Publisher’s Note

All claims expressed in this article are solely those of the authors and do not necessarily represent those of their affiliated organizations, or those of the publisher, the editors and the reviewers. Any product that may be evaluated in this article, or claim that may be made by its manufacturer, is not guaranteed or endorsed by the publisher.

## Footnotes

1
http://www.R-project.org
